# Staatsdoping im DDR-Leistungssport: Gesundheitliche Folgen, Aufarbeitung und rechtlicher Ausgleich

**DOI:** 10.1007/s00103-026-04238-2

**Published:** 2026-04-27

**Authors:** Carsten Spitzer, Burkhard Bley

**Affiliations:** 1 Klinik und Poliklinik für Psychosomatische Medizin und Psychotherapie, Universitätsmedizin Rostock, Gehlsheimer Straße 20, 18147 Rostock, Deutschland; 2 Landesbeauftragter für Mecklenburg-Vorpommern für die Aufarbeitung der SED-Diktatur, Schwerin, Deutschland

**Keywords:** DDR-Leistungssportsystem, Staatliches Zwangsdoping, SED-Unrecht, Komplexe Sportschäden, Gesellschaftliche Aufarbeitung, GDR competitive sports system, State-imposed forced doping, SED injustice, Complex sports impairments, Societal reappraisal

## Abstract

Vor dem Hintergrund der Systemkonkurrenz zwischen Kommunismus und Kapitalismus wurde der Sport in der DDR politisch instrumentalisiert. Dies zeigte sich unter anderem in einem staatlich gelenkten, kontrollierten, hierarchisch organisierten und in Teilen totalitären Leistungssportsystem. Dessen zentraler Bestandteil war ein konspirativ geplantes, staatlich gesteuertes Zwangsdoping, das ab den 1970er-Jahren systematisch umgesetzt wurde und schätzungsweise bis zu 15.000 minderjährige Athletinnen und Athleten betraf. Leistungssteigernde Substanzen, vor allem anabolandrogene Steroide, wurden verdeckt, ohne Wissen, Aufklärung oder Einwilligung der Betroffenen verabreicht, häufig vor der Pubertät und trotz bekannter gesundheitlicher Risiken. Zu den Folgen der Dopingmittel, die Trainingsbelastungen jenseits physiologischer Grenzen ermöglichten, kamen Disziplinierung, Leistungszwang, Überwachung, Abschottung und Missbrauch durch Sportfunktionäre hinzu. Die daraus resultierenden Beeinträchtigungen der biopsychosozialen Entwicklung sind bis heute in Form schwerer gesundheitlicher Schäden nachweisbar. Neben Störungen hormoneller Regelkreise, Wachstums- und Organschäden, Herz-Kreislauf-Erkrankungen, Tumoren sowie degenerativen Erkrankungen des Bewegungsapparats stehen psychische Störungen mit einer Lebenszeitprävalenz von bis zu 98 % im Vordergrund, insbesondere depressive, phobische und somatoforme Schmerzstörungen. Trotz der Dopingopfer-Hilfegesetze bleibt die politisch-juristische Aufarbeitung dieses durch die SED begangenen Unrechts unzureichend. Auch gesellschaftlich wird bislang zu selten anerkannt, dass die minderjährig zwangsgedopten ehemaligen Leistungssportlerinnen und -sportler in ihren Menschenrechten verletzt wurden und bis heute unter schweren körperlichen und psychosozialen Folgeschäden leiden.

## Abstract (en)

Against the backdrop of the ideological rivalry between communism and capitalism, sport in the GDR was exploited for political ends. This was evident, among other things, in a state-directed, controlled, hierarchically organized and, in parts, totalitarian competitive sports system. A key component was a conspiratorial, state-controlled program of compulsory doping, which was systematically implemented from the 1970s onwards and affected an estimated 15,000 underage athletes. Performance-enhancing substances, primarily anabolic-androgenic steroids, were administered covertly, without the knowledge, information, or consent of those affected, often before puberty and despite known health risks. In addition to the effects of the doping substances, which enabled training loads beyond physiological limits, there was discipline, pressure to perform, surveillance, isolation, and abuse by sports officials. The resulting impairments to biopsychosocial development are still evident today in the form of serious health damage. Alongside disruptions to hormonal regulatory systems, growth and organ damage, cardiovascular diseases, tumors and degenerative diseases of the musculoskeletal system, and mental disorders with a lifetime prevalence of up to 98% are of particular concern, especially depressive, phobic, and somatoform pain disorders. Despite the laws providing assistance to victims of doping, the political and legal management of this particular injustice perpetrated by the SED remains unsatisfactory. Furthermore, society rarely acknowledges the violation of the human rights of former competitive athletes in the GDR who were forcibly doped as minors and continue to suffer from severe physical and psychosocial consequences to this day.

## Einleitung

Die Deutsche Demokratische Republik (DDR) war trotz ihrer relativ geringen Einwohnerzahl mit 577 olympischen Medaillen sowie 768 Welt- und 747 Europameistertiteln eine der erfolgreichsten Sportnationen ihrer Zeit [1]. Diese Erfolge des „Sportwunderlandes“ beruhen auf verschiedensten Faktoren, allen voran einer rigorosen politischen Instrumentalisierung, die zur Entwicklung eines ausgefeilten Leistungssportsystems, inklusive eines staatlichen Zwangsdopings mit deletären gesundheitlichen (Langzeit‑)Folgen bei den Athletinnen und Athleten, führte [1–6]. In diesem Beitrag werden zunächst die zeitgeschichtlich-politischen Hintergründe und das Leistungssportsystem der DDR vorgestellt, um dann das seit Mitte der 1970er-Jahre staatlich zentralisierte, gesteuerte, konspirative und flächendeckende Zwangsdoping zu skizzieren. Anschließend werden schlaglichtartig die bis heute anhaltenden, schweren körperlichen und psychosozialen Folgeschäden von minderjährig zwangsgedopten ehemaligen Leistungssportlerinnen und -sportlern beleuchtet. Schließlich werden die gesellschaftliche Aufarbeitung und der Umgang mit dieser spezifischen Form von SED-Unrecht dargestellt.

## Die zeitgeschichtlich-politischen Hintergründe

Sport, insbesondere der Leistungssport, hat seit jeher eine politische Dimension. Die politisch-ideologische Vereinnahmung des DDR-Leistungssports durch die Machthaber und die Sozialistische Einheitspartei Deutschlands (SED) kann nur im Kontext des Kalten Krieges und der Systemkonkurrenz zwischen Kapitalismus und Kommunismus verstanden werden [7]. Diese Vereinnahmung war insofern besonders, als dass Manipulation, Indoktrination und Überwachung durch das Ministerium für Staatssicherheit (MfS) den Leistungssport prägten [8, 9]. Er stellte aufgrund seiner Isolation und des hohen Grads an Kontrolle ein nahezu totalitäres System dar, in dem Menschenrechte missachtet wurden [8–10]. Er war der einzige Bereich innerhalb der sozialistischen Gesellschaft, in dem das kapitalistische Wettbewerbsprinzip konsequent zur Anwendung kam [11].

Der DDR-Leistungssport erfüllte 2 Funktionen: Er stellte eines der wichtigsten Propagandainstrumente dar, um die Überlegenheit des Sozialismus gegenüber dem Kapitalismus auszuweisen, und sicherte so Macht- und Herrschaftsansprüche innerhalb dieser Systemkonkurrenz ab. Dabei sollten auch politische, ökonomische und soziale Defizite verdeckt werden – sowohl nach außen als auch nach innen. Zudem fungierten die Athletinnen und Athleten als Idole, repräsentierten gewissermaßen den Prototyp der sozialistischen Persönlichkeit und dienten somit als Identifikationsfiguren für die eigene Bevölkerung [2, 11, 12]. In Bezeichnungen wie „Turnschuhdiplomaten“ oder „Diplomaten im Trainingsanzug“ klingt die politische Dimension des DDR-Leistungssportes an; gleichzeitig zeigt sich hier eine euphemistische Verharmlosung: Es ging eben nicht darum, die Interessen der beteiligten Sportlerinnen und Sportler zu erkennen, zu respektieren und diese im Rahmen eines Aushandlungsprozesses zu würdigen, was als zentrales Merkmal der Diplomatie gilt.

Nach dem Ende der nationalsozialistischen Herrschaft musste in der 1949 gegründeten DDR der Sport grundsätzlich neu organisiert werden. Dabei erwiesen sich die vielfältigen Versuche in den 1950er- und 1960er-Jahren, den prestigeträchtigen DDR-Spitzensport repräsentativer zu gestalten, ihn zu strukturieren und ein zentral steuerndes Komitee unter politisch geführter Hand zu entwickeln, als wenig erfolgreich [2, 3, 13, 14]. Insbesondere mit Blick auf die olympischen Spiele 1972 in München, bei denen die DDR erstmals mit eigener Fahne und eigener Nationalhymne startete,^1^ wurden Ende der 1960er-Jahre jene Strukturen geschaffen, die effektiv und nachhaltig den Erfolg sichern sollten. So formuliert Ritter [14, S. 257]: „Erst die im Herbst 1967 vorherrschende Konstellation bestimmter Voraussetzungen im Zusammenhang mit den entscheidenden Erkenntnissen aus den [Strukturierungsfehlversuchen in den 1950er- und 1960er-Jahren] brachten das [Führungs- und] Leistungsmodell zustande, das die 1970er und 1980er-Jahre prägte.“

Im Frühjahr 1969 erließ das Zentralkomitee der SED den sogenannten Leistungssportbeschluss (offiziell: „Grundlinie der Entwicklung des Leistungssports in der DDR bis 1980“), der nicht nur Leistungsvorgaben für Olympia 1972 festlegte, sondern auch die Förderung nach dem Kosten-Nutzen-Prinzip. Das bedeutete konkret eine Priorisierung medaillenstarker olympischer Disziplinen, vor allem Einzelsportarten mit hoher Medaillenausbeute auf Kosten von personal- und kostenintensiven Mannschaftssportarten. Der Wegfall der Förderung und die damit verbundenen Einschnitte in manchen Disziplinen beendeten Hunderte Karrieren [2, 15]. Zugleich wurde durch die Auswahl bestimmter Sportarten deren Leistungsniveau deutlich erhöht [15]. Der Leistungssportbeschluss umfasste zudem Maßnahmen der Strukturierung und Zentralisierung, etwa den Ausbau von Kinder- und Jugendsportschulen (KJS), Sportclubs (SC) oder Forschungseinrichtungen wie das Forschungsinstitut für Körperkultur und Sport (FKS), das in der Dopingforschung eine führende Rolle spielte [1, 14, 16, 17]. Schließlich wurden neben der Steuerung durch die SED auch Geheimhaltung respektive strenge Vertraulichkeit beschlossen.

## Das Leistungssportsystem der DDR

Auf der institutionellen Ebene war und blieb das Politbüro der SED das entscheidende Gremium. Für die Umsetzung und Kontrolle waren unter anderem die Abteilung Sport des Zentralkomitees der SED sowie die Staats- und Ministerräte zuständig [1, 18]. Die zentralen staatlichen Instanzen waren das Staatssekretariat für Körperkultur und Sport (StKS) und die Leistungssportkommission (LSK). Das Staatssekretariat koordinierte den Sportbetrieb, förderte Forschung und sorgte für die Zielerreichung der zentral vorgegebenen Pläne. Die LSK organisierte die Zusammenarbeit zwischen allen Funktionären, plante und koordinierte die Forschung und entschied über Leistungsvorgaben, die in den Jahresplänen festgeschrieben wurden [1, 18]. Der Deutsche Turn- und Sportbund (DTSB) war als Dachorganisation des DDR-Sports für die gesellschaftlichen Strukturen zuständig. Eine staatlich gelenkte sportwissenschaftliche Forschung erfolgte zu vielfältigsten Themenfeldern (unter anderem Biomechanik, innovative Trainingsmethoden) und wurde ganz wesentlich am FKS der Deutschen Hochschule für Körperkultur in Leipzig vorangetrieben [16].

Ein Ergebnis der Aktivitäten dieser Institutionen war die Einführung des „Einheitlichen Sichtungs- und Auswahlsystems“ (ESA) im Jahr 1973. Damit sollten zielgerichtet junge Talente für den Leistungssport gewonnen werden. Das ESA-System erfasste fast alle Kinder im Vorschul- und Schulalter und hatte Züge einer Totalerhebung; nach einer ersten Leistungsprüfung wurden ausgewählte Kinder in das pyramidal aufgebaute Leistungssportsystem mit seinen 3 Begabtenförderstufen integriert [18, 19]. Dabei bestimmten nicht die kindlichen Vorlieben, sondern vielmehr deren körperliche Voraussetzungen die Sportart.

Die Basis der Kaderpyramide bildeten als erste Förderstufe die Trainingszentren, in die man nur nach erfolgreicher Leistungsprüfung delegiert wurde. Die zweite Förderstufe bestand aus den KJS, die systematisch auf- und ausgebaut wurden. In der höchsten Förderstufe waren die Hochleistungssportlerinnen und -sportler und Mitglieder der Nationalmannschaften. Hervorzuheben ist die Institution der KJS, weil hier die Lebenswelt der Kinder und Jugendlichen nicht bloß umfassend auf den Sport ausgerichtet war, sondern auch von den erwachsenen Akteuren minutiös geplant und kontrolliert wurde [4, 5, 17, 20, 21]. Die Delegation zur KJS erfolgte in manchen Sportarten bereits ab der 1. Klasse, in anderen ab der 5. oder 8. Klasse und war meist mit einer Internatsunterbringung, weit entfernt vom Elternhaus, verbunden. Die Abschottung dieses Systems ergab sich auch aus der unmittelbaren Nähe von KJS und Trainingsstätte und der Zugang zu dem Gelände war häufig nur über eine Ausweiskontrolle möglich. Die Beschulung wurde zugunsten des Trainings flexibilisiert, etwa durch eine Streckung von 2 auf 3 Schuljahre [17, 19, 21]. Der Alltag in der KJS war von immensen Trainingspensen mit bis zu 30 h pro Woche und regelmäßiger Überschreitung der körperlichen und seelischen Leistungsgrenzen, Disziplinierung, politischer Einflussnahme und Abschottung nach außen gekennzeichnet [4, 5, 8, 21]. Die minderjährigen Athletinnen und Athleten wurden in diesem strengen Regime häufig mit Verheimlichungs- und Schweigegeboten zu sämtlichen Praktiken belastet, die die Kommunikation zum Elternhaus und dem familiären Umfeld erheblich belasteten [8, 10, 17, 22].

## Staatliches Zwangsdoping

Diese Maßnahmen, einschließlich relevanter Innovationen, mussten vor den Blicken des „Klassenfeindes“ und weiterer Konkurrenz verborgen bleiben, sodass das Leistungssportsystem zu einem Staatsgeheimnis avancierte, welches mithilfe des MfS geschützt werden musste [8, 9]. Dazu zählte ab den frühen 1970er-Jahren auch die konspirative Dopingpraxis, die spätestens seit der Verabschiedung des „Staatsplans 14.25“ (im Jargon auch als „Staatsplan Sieg“ bezeichnet) 1974 staatlich zentralisiert und gesteuert sowie flächendeckend erfolgte und nach Schätzungen bis zu 15.000, meist minderjährige Leistungssportlerinnen und -sportler betraf [23].

Bereits Anfang der 1960er-Jahre experimentierten Trainingszentren und Sportclubs mit Aufputschmitteln, um ab 1964 auf Initiative des FKS dezentral, aber systematisch Anabolika zu erproben [3–5, 20, 21]. Ab Ende der 1960er-Jahre kam es angesichts der zunehmenden Konkurrenz und gehäufter Wettkampf- und Trainingskontrollen zu einer staatlichen Zentralisierung von Einkauf, Distribution, Anwendung und Beforschung der Dopingmittel, wobei der VEB Jenapharm eine wichtige Rolle spielte [21, 24]. Diese Systematisierung wurde mit dem Staatsplan 14.25 festgeschrieben und ausgebaut. Von nun an erfolgte unter Missachtung rechtlicher, ethischer und moralischer Prinzipien die Vergabe von „unterstützenden Mitteln“ (uM), wie sie verschleiernd genannt wurden. Die am häufigsten verwendeten Dopingsubstanzen in der DDR waren anabolandrogene Steroide (AAS), besonders Dehydrochlormethyltestosteron, das unter dem Handelsnamen Oral-Turinabol (OT) auch als Arzneimittel zugelassen war und Betroffenen als „blaue Pille“ bekannt ist. Doch auch andere Substanzen wie Neuropeptide, Psycho- und Nootropika, Stimulanzien und Schmerzmittel sowie Blutbestrahlungen wurden im geheimen Staatsdoping missbräuchlich eingesetzt [3–5, 20, 25, 26].

Die oben erwähnten Gremien wie LSK und DTSB, die im engen Austausch mit der SED-Staatsführung standen, erließen im 4‑jährigen Zyklus konspirative Richtlinien zur Anwendung und Kontrolle der „uM“, auf denen die jährlich neu formulierten Anwendungskonzeptionen fußten. Diese enthielten Vorgaben für das jeweilige Folgejahr, z. B. technische und taktische Anforderungen an die Athletinnen und Athleten, aber vor allem Art und Dosis der Dopingmittel. Deren Vergabe erfolgte ohne Aufklärung über Wirkung und Nebenwirkungen respektive eine informierte Einwilligung der Betroffenen und/oder ihrer Eltern. Vielmehr wurden die „uM“ als Eiweiße bzw. Vitamine ausgegeben und getarnt (z. B. in das Essen gemischt) oder in Form von Schokolade, Kontrazeptiva oder Nasensprays verabreicht [3–5, 20, 25, 26]. Eine Verweigerung der Einnahme von „uM“ war praktisch nicht möglich, weil in solchen Fällen sofort gedroht wurde (z. B. mit negativen Konsequenzen für die eigene Ausbildung).

Während offiziell der Dopingbeginn ab dem 16. Lebensjahr vorgesehen war, wurde das Merkmal der „biologischen Reife“ zusätzlich in die Richtlinien aufgenommen, das eine dehnbare Definition des Mindestalters zur Erstanwendung von Doping zuließ [3–5, 22]. Für Sportarten mit einem niedrigeren Höchstleistungsalter (etwa Turnen) konnte damit die Altersgrenze für die Dopingvergabe deutlich herabgesetzt werden und begann zum Teil im Grundschulalter [3, 21, 22]. Infolgedessen wurden minderjährigen Sportlerinnen und Sportlern, die weit vor der biologischen Reife standen, regelmäßig und kontinuierlich übermäßig hohe Dosen unerlaubter Substanzen verabreicht, was ein Training weit über die physiologischen Leistungsgrenzen hinaus ermöglichte – mit desaströsen Folgen für die Entwicklung und Gesundheit der Betroffenen [6, 20, 22, 26–31]. Den verantwortlichen Sportfunktionären waren die deletären und zum Teil irreversiblen Folgen des Dopings – gerade bei Minderjährigen und jungen Erwachsenen – nicht nur bekannt, sondern sie wurden von ihnen billigend in Kauf genommen. Zu betonen ist ebenfalls, dass der missbräuchliche Einsatz von Arzneimitteln in der DDR offiziell verboten war – der Staat warb für einen „sauberen“ Sport. Das geheime Doping stellte somit einen Verstoß gegen die eigenen Gesetze dar, sodass die Abschirmung durch das MfS nicht nur nach außen, sondern auch nach innen zwingend erforderlich war; der gesamte DDR-Leistungssport war von einem komplexen Geheimdienstnetzwerk durchdrungen [8, 9].

## Gesundheitliche Langzeitfolgen bei minderjährig zwangsgedopten ehemaligen Leistungssportlern

Die biomedizinisch geprägte Forschung hat sehr auf die körperlichen Folgen des Staatsdopings fokussiert [2, 3, 6, 20, 30], wobei Schäden in einem zeitlichen Zusammenhang mit der Einnahme von „uM“ bereits in zeitgenössischen Berichten des MfS dokumentiert wurden, unter anderem irreversible Veränderungen der Geschlechtshormonachsen mit Wachstumsstörungen, Hautveränderungen, Herz-Kreislauf-Störungen und Lebererkrankungen, inklusive Tumoren [2–6, 25, 26]. Nach der Wiedervereinigung ergänzten Einzelfalluntersuchungen im Zuge der juristischen und wissenschaftlichen Aufarbeitung des DDR-Leistungssports diese Liste um gehäuftes Auftreten von Tumoren, Wachstumsstörungen der Geschlechtsorgane bei beiden Geschlechtern, erhöhtes Risiko für Fehlgeburten und Unfruchtbarkeit bei Frauen und degenerative Erkrankungen des Skelettsystems. Letztere resultieren aus der unphysiologisch hohen und altersunangemessenen Trainingsbelastung, welche durch die Einnahme von AAS erst ermöglicht wurde [27] und häufig mit bis heute anhaltenden Schmerzsyndromen und der Notwendigkeit späterer Gelenkersatzoperationen verbunden ist [31]. Studien, die Betroffene entweder direkt befragten oder Facharzt‑, Krankenhaus- sowie Rehabilitationsberichte analysierten, geben die Häufigkeit orthopädischer Erkrankungen mit über 90 % an [6, 30]. Aus Begutachtungsuntersuchungen ehemaliger DDR-Leistungssportlerinnen und -sportler sind folgende Häufigkeiten für Krankheiten des Skelettsystems bekannt: Bei insgesamt 75 % der Befragten bestehen Arthrosen; 25 % leiden unter Bandscheibenschäden und 21 % unter Meniskusschäden [27]. Zum Vergleich: In der deutschen Allgemeinbevölkerung leiden 28,7 % der 50- bis 59-jährigen Personen unter Arthrose und dieser Anteil steigt auf maximal 42,4 % in der Gruppe der über 70-Jährigen [32]. In einer Studie in Kooperation mit dem Doping-Opfer-Hilfeverein (DOH) berichten 25 % der Betroffenen von Herzerkrankungen, 27 % von Krebserkrankungen und 27 % der befragten Frauen von gynäkologischen Schädigungen [28]. Zu sehr ähnlichen oder sogar höheren Prävalenzen kommen auch andere Studien [6, 30].

Während die körperlichen Gesundheitsschäden ganz überwiegend als direkte oder indirekte Folge des Zwangsdopings der damals minderjährigen Athletinnen und Athleten begriffen werden [6, 20, 25, 26], erscheint die Sachlage hinsichtlich psychischer und psychosozialer Folgeschäden deutlich komplexer, weil viele weitere Aspekte des politisch instrumentalisierten und ideologisch gerahmten DDR-Leistungssportsystems pathogene Potenz hatten [22, 33]. Dazu können unter anderem die relative Isolation, die frühe Ablösung und Entfremdung vom Elternhaus, die ausschließliche Fokussierung auf den Sport, einschließlich der immensen Trainingsumfänge, sowie der daraus resultierende Verzicht auf andere alterstypische Interessen, der meist wenig liebevolle Umgang der Funktionäre mit den Sportlern, Disziplinierung, politische Einflussnahme und der unbedingte Leistungszwang zählen [6, 10, 17]. Besonders relevant erscheinen in diesem Kontext jedoch emotionaler Missbrauch (z. B. schwere Demütigungen und Beschimpfungen), aber auch körperliche Gewalt und sexuelle Grenzverletzungen, von denen viele Betroffene berichten [27, 28]. In einer aktuellen Studie berichteten 55,9 % der befragten ehemaligen Leistungssportlerinnen und -sportler von emotionalem Missbrauch durch ihre Trainer, gefolgt von körperlicher (49,5 %) und sexueller Gewalt (22,6 %; [34]).

Vor diesem Hintergrund verwundert es nicht, dass in allen bisherigen Untersuchungen die Mehrheit von psychischen „Problemen“ respektive Störungen betroffen war bzw. ist [27, 28], wobei die Angaben zwischen 62 % [6] und 73 % [30] schwanken. Typische Störungen, die bei ehemaligen DDR-Kaderathletinnen und -athleten signifikant häufiger als in der Allgemeinbevölkerung vorkommen, sind depressive Erkrankungen, Anpassungs-, posttraumatische Belastungs-, somatoforme Schmerz- sowie Essstörungen [6, 27, 29, 30]. Die jüngste und methodisch hochwertigste Studie berichtet gar von einer Lebenszeitprävalenz irgendeiner psychischen Störung von 98 % bei minderjährig zwangsgedopten Spitzensportlerinnen und -sportlern [33]. Mittels eines standardisierten Interviews wurden sowohl im Quer- als auch im Längsschnitt Angst-, depressive und somatoforme Schmerzstörungen am häufigsten diagnostiziert. Innerhalb der Angststörungen waren spezifische Phobien vom Spritzen- und Verletzungstypus besonders häufig vertreten (Lebenszeitprävalenz von 66,4 %). Die hohe Lebenszeitprävalenz der somatoformen Schmerzstörung von 76,2 % korrespondiert mit den Erfahrungen der Betroffenen im Leistungssport [33]. Dabei ist zu berücksichtigen, dass ihre psychischen Beeinträchtigungen bis heute anhalten, denn sie waren bei den jeweiligen Untersuchungszeitpunkten deutlich depressiver als die Allgemeinbevölkerung [29, 33]. Gerade bei den depressiven Störungen lässt sich innerhalb des komplexen Bedingungsgefüges multipler biopsychosozialer Faktoren die pathogene Relevanz von AAS plausibilisieren: Die Häufigkeit von Depressionen ist bei Leistungssportlern vergleichbar mit derjenigen in der Allgemeinbevölkerung [35], aber bei Athleten mit AAS-Gebrauch generell, d. h. auch ohne den Kontext von Zwang, deutlich höher [36].

## Gesellschaftliche Aufarbeitung und Fragen eines rechtlichen Ausgleichs

Seit den 1970er-Jahren wurde in der westdeutschen Presse über „Drill und Heuchelei“ sowie Doping im DDR-Spitzensport diskutiert – anhand von Aussagen geflüchteter Sportler, aber auch wegen körperlicher Auffälligkeiten. Auf die Frage nach den tiefen Stimmen der DDR-Schwimmerinnen bei den olympischen Spielen 1976 in Montreal antwortete Trainer Rolf Gläser: „Die sind zum Schwimmen hier und nicht zum Singen“ [2, 37, 38]. Eine Aufarbeitung des DDR-Sportsystems konnte erst nach dem Sturz der SED-Diktatur und der Öffnung der Archive stattfinden. Wichtige Impulse setzten Brigitte Berendonk und Werner W. Franke mit ihren 1991 veröffentlichten Forschungen und Franke mit seiner Strafanzeige gegen verantwortliche DDR-Sportfunktionäre. Auf Grundlage der Ermittlungen der Zentralen Ermittlungsstelle Regierungs- und Vereinigungskriminalität (ZERV) wurden seit 1998 in 38 Verfahren 67 Beschuldigte angeklagt und 49 Beklagte verurteilt, davon 17 zu Freiheitsstrafen auf Bewährung. Angesichts der quantitativen und moralischen Dimension des Unrechts im DDR-Sport können die Ergebnisse der unter dem Zeitdruck der Verjährung im Oktober 2000 geführten juristischen Aufarbeitung nicht überzeugen [22, 39], eher durch den Erkenntnisgewinn und die öffentliche Aufmerksamkeit. Der DDR-Sport war mehrfach Untersuchungsgegenstand parlamentarischer Gremien und Anhörungen, so in der Enquetekommission des Bundestags zur SED-Diktatur 1992 bis 1994 oder im Landtag Mecklenburg-Vorpommern 2015/2016 [25, 40, 41].

Öffentlich verhandelt werden gegenwärtig konträre Zuschreibungen für ehemalige DDR-Spitzensportler: einerseits die unkritische, nostalgische Sicht auf die sportlichen Helden des untergegangenen Landes, deren Erfolge man bewundert und die man sich nicht auch noch wegnehmen lassen will, andererseits die Sicht auf überzeugte oder zumindest hochgradig angepasste „Diplomaten im Trainingsanzug“ als verachtete oder beneidete Nutznießer des DDR-Systems. Gesellschaftlich noch wenig etabliert ist das Narrativ einer Betroffenengruppe, die durch die rücksichtlose Instrumentalisierung für die Zwecke des SED-Staats in ihren Menschenrechten verletzt wurde und heute unter den körperlichen und seelischen Folgen leidet. Die meisten prominenten Spitzensportler zeigen sich nicht solidarisch gegenüber den weniger erfolgreichen Trainingskameraden aus den unteren Regionen der Kaderpyramide und schweigen beharrlich oder leugnen die Schattenseiten des DDR-Sports. Die Deutungshoheit über den DDR-Sport ist umkämpft wie bei kaum einem anderen DDR-Thema. Streit herrscht nicht nur – wie zu erwarten – zwischen Kritikern und Rechtfertigern des DDR-Sports. Im Zusammenhang mit dem 2. Dopingopfer-Hilfegesetz wurde über die Zahl der Berechtigten, über Kriterien und Betrugsmöglichkeiten diskutiert, aber auch zu den Fragen, ob man sich als Sportler oder Trainer dem Zwangsdoping entziehen konnte oder ob nachfolgende Generationen geschädigt sein könnten. Wenig hilfreich für die Aufarbeitung des DDR-Sports waren die nicht immer sachliche Form der Auseinandersetzung, persönliche Diskreditierungen, eine mitunter qualitativ fragwürdige mediale Begleitung und eine Flut von Beiträgen über digitale Kanäle, welche gesicherte Erkenntnisse über den DDR-Sport infrage stellten [42–45].

Auf der Grundlage von 2 Dopingopfer-Hilfegesetzen erhielten zwischen 2002 und 2007 bzw. 2016 bis 2019 befristet 1643 ehemalige DDR-Sportler einen einmaligen finanziellen Ausgleich in Höhe von 10.500 € für die gesundheitlichen Folgen der ihnen unwillentlich oder unwissentlich verabreichten Dopingmittel [46]. Die Behörde des Landesbeauftragten für Mecklenburg-Vorpommern (M-V) begleitete seit 2016 insgesamt etwa 350 Antragsteller in diesen Verfahren sowie später bei der Beantragung einer verwaltungsrechtlichen Rehabilitierung als Voraussetzung für die Inanspruchnahme von Leistungen des sozialen Entschädigungsrechts. Für Betroffene, die als Sportler in der DDR auf dem Gebiet des heutigen Landes M‑V geschädigt wurden, öffnete das Verwaltungsgericht Greifswald mit seinem Urteil vom 28.12.2020 den Weg zur Anerkennung nach dem Verwaltungsrechtlichen Rehabilitierungsgesetz (VwRehaG; [47]). Neben den insgesamt 51 Rehabilitierungen in M‑V waren ehemalige DDR-Sportler nur in Sachsen und Thüringen in wenigen Fällen erfolgreich.

In der Revision gegen eine Ablehnung durch das Verwaltungsgericht Potsdam entschied das Bundesverwaltungsgericht am 27.03.2024, dass DDR-Sportgeschädigte für die gesundheitlichen Folgen des in der DDR an ihnen vorgenommenen systematischen staatlichen Dopings keine verwaltungsrechtliche Rehabilitierung in Anspruch nehmen können [48]. Zwar wäre das staatliche Zwangsdoping als Maßnahme einzustufen, die „in schwerwiegender Weise gegen die Prinzipien der Gerechtigkeit, der Rechtssicherheit oder der Verhältnismäßigkeit verstoßen“ [49] habe. Es erfülle aber nicht das weitere gesetzliche Erfordernis einer politischen Verfolgung oder eines Willkürakts im Einzelfall. Das Gericht stützt die geläufige Zuschreibung für DDR-Sportler, die als privilegierte Exponenten des SED-Staats trotz politischer Instrumentalisierung nicht Opfer von SED-Unrecht seien. Eine Benachteiligung gegenüber einer vergleichbaren Personengruppe sei nicht feststellbar [48, 49]. Für DDR-Sportgeschädigte ist nach dieser Entscheidung derzeit keine Anerkennung nach VwRehaG mehr möglich.

Bereits erteilte Rehabilitierungsbescheide bleiben gültig, sodass Verfahren nach dem Bundesversorgungsgesetz bzw. dem seit 2024 gültigen Sozialgesetzbuch (SGB) XIV in Verbindung mit dem VwRehaG weitergeführt werden können. Von den in der Beratung beim Landesbeauftragten M‑V betreuten Betroffenen ist lediglich in einem Fall bekannt, dass ein Grad der Schädigungsfolgen (GdS) von mehr als 30 zuerkannt wurde, der mit einer monatlichen Entschädigungszahlung verbunden ist. Je nach Höhe des GdS sind in der Sozialen Entschädigung monatliche Leistungen zwischen 434 € bis zu 2169 € möglich. DDR-Sportgeschädigte scheitern regelmäßig an den Beweisanforderungen der Versorgungsverwaltungen. Ohne Nachweise über konkrete Medikationen, verwendete Substanzen, Dosierungen und Zeitspannen wird eine Kausalität zu den geltend gemachten gesundheitlichen Schäden nicht anerkannt. Diese sind aufgrund der konspirativen Praxis der Vergabe von Doping- und anderen Arzneimitteln im DDR-Leistungssport kaum zu erbringen. Einer vergleichbaren Beweisnot in den Verfahren unterliegen bisher^2^ auch andere Betroffene von SED-Unrecht wie ehemalige politische Häftlinge, Opfer von Zersetzungsmaßnahmen oder Umerziehung in Spezialheimen.

Zudem greift aus Sicht der Beratungspraxis die Überbetonung der pharmakologischen Aspekte bei der versorgungsmedizinischen Prüfung der Gesundheitsfolgen angesichts der schwerwiegenden und komplexen körperlichen und seelischen Schädigungen der ehemaligen Sportler zu kurz. Wie oben dargestellt, lebten die jungen Sportlerinnen und Sportler in einem fast hermetisch abgeschlossenen Zwangssystem, das Merkmale einer totalen Institution aufwies, deren negative Auswirkungen hinlänglich bekannt sind [50]. In Kombination mit dem Zwangsdoping und eventuellen Missbrauchserfahrungen ist es daher folgerichtig, von „komplexen Sportschäden“ bzw. „Sportgeschädigten“ zu sprechen [5, 16].

In das Gesetzespaket der im Januar 2025 vom Bundestag verabschiedeten verbesserten rechtlichen Regelungen für Betroffene von SED-Unrecht haben die DDR-Sportgeschädigten keine Aufnahme gefunden. Zeitgleich wurde vom Bundestag eine Entschließung beschlossen, in der eine Anerkennung und Unterstützung der Geschädigten des staatlich organisierten Dopingsystems der ehemaligen DDR gefordert wurde. Diese Forderung hat Eingang in den Koalitionsvertrag der aktuellen Bundesregierung aus CDU und SPD gefunden und harrt noch der Umsetzung.

In der Beratung von DDR-Sportgeschädigten gibt es besondere Herausforderungen. Der mit der Doping-Aufarbeitung wahrgenommene Vertrauensbruch von engen Bezugspersonen hat Folgen für die Beziehungsfähigkeit und führt zu erhöhtem Misstrauen der Betroffenen. Wer auf Leistungsanspruch konditioniert ist, dem fällt es schwer, gesundheitliche Schäden und Verfahrenshindernisse nicht als Kränkung und Niederlage zu begreifen. Mit Scham verbunden ist das Eingeständnis, für ein System missbraucht worden zu sein und Hilfe annehmen zu müssen. Die Dauer der Verfahren über Jahre, Nachweispflichten, Begutachtungen, Ablehnungsbescheide, Widersprüche, Klagen vor Gericht sind für Betroffene zermürbend, Aufgeben ist aber meist keine Option [48].

## Fazit

Für die eigennützigen Ziele des SED-Staates, um das Ansehen der DDR zu erhöhen, wurden minderjährig zwangsgedopte Sportlerinnen und Sportler durch eine rücksichtlose Instrumentalisierung nicht nur in ihrer Menschenwürde verletzt, sondern auch in ihrer biopsychosozialen Entwicklung geschädigt. Sie leiden bis heute nicht nur an den physischen, sondern auch und gerade an den chronischen psychischen und sozialen Folgenschäden, die ihre Lebensqualität stark reduzieren. Das Unrecht und die in der Folge erlittenen komplexen Schädigungen sollten durch eine gesetzliche Regelung anerkannt werden. Betroffene, die aufgrund des Unrechts in ihrer beruflichen und gesellschaftlichen Teilhabe erheblich beeinträchtigt sind, sollten einen angemessenen, dauerhaften und regelmäßigen finanziellen Ausgleich erhalten. Zu diesem Zweck könnte der Gesetzgeber das Verwaltungsrechtliche Rehabilitierungsgesetz entsprechend ergänzen und die Verordnung zur Vermutung des ursächlichen Zusammenhangs bestimmter schädigender Ereignisse mit bestimmten gesundheitlichen Schädigungen anpassen.

## Footnotes

1 Sportlerinnen und Sportler der DDR nahmen von 1956 bis 1964 an Olympia als Teil einer gesamtdeutschen Mannschaft teil, die unter der Olympiafahne mit den 5 Ringen antrat. Die DDR trat erstmals 1968 mit einer eigenen Olympiamannschaft als Ost-Deutschland auf, allerdings weiterhin unter einer gemeinsamen Fahne mit der bundesdeutschen Mannschaft (Schwarz-Rot-Gold mit den olympischen Ringen).

2 Mit dem am 01.07.2025 in Kraft getretenen Sechsten Gesetz zur Verbesserung rehabilitierungsrechtlicher Vorschriften für Opfer der politischen Verfolgung in der ehemaligen DDR wurde zur Beweiserleichterung bei der Anerkennung verfolgungsbedingter Gesundheitsschäden u. a. im Strafrechtlichen und Verwaltungsrechtlichen Rehabilitierungsgesetz eine kriterienbasierte Vermutungsregelung eingeführt. Seine Wirkung kann diese Änderung beim Vollzug im sozialen Entschädigungsrecht erst durch den noch ausstehenden Erlass einer entsprechenden Rechtsverordnung entfalten.
